# A Helping Hand: RNA-Binding Proteins Guide Gene-Binding Choices by Cohesin Complexes

**DOI:** 10.1371/journal.pgen.1006419

**Published:** 2016-11-17

**Authors:** Alyssa N. Coyne, Daniela C. Zarnescu

**Affiliations:** Departments of Molecular and Cellular Biology, Neuroscience and Neurology, University of Arizona, Tucson, Arizona; Geisel School of Medicine at Dartmouth, UNITED STATES

**Fig 1:**
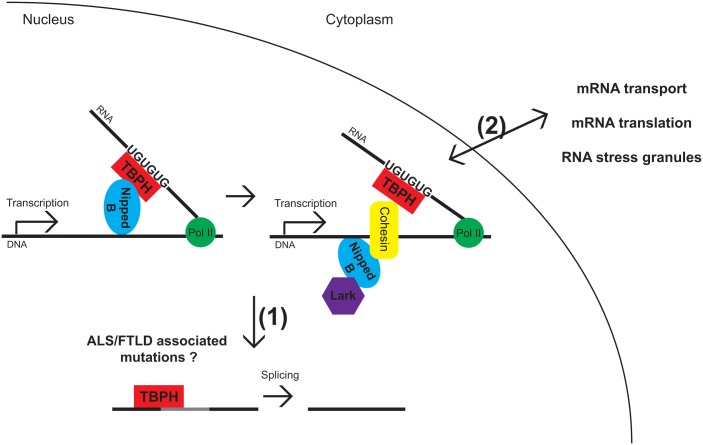
Model for TBPH and Lark interacting with Nipped-B and cohesin. TBPH binds to UG-rich sequences on nascent transcripts. This recruits Nipped-B, which in turn recruits cohesin and Lark to DNA. TBPH also participates in the splicing of newly transcribed RNAs. In future work it will be interesting to determine how nuclear depletion of TDP-43 or disease-associated mutations affect the coupling of transcription regulation by cohesion/Nipped-B and RNA processing, whether it is (1) splicing or (2) mRNA transport, translation, or association with RNA stress granules. It also remains to be determined whether TBPH/TDP-43 associates with the same RNA targets in all steps of RNA metabolism.

Cohesin complexes have been extensively studied for their roles in sister chromatid cohesion during cell division, and in addition, regulate transcription through multiple mechanisms. Together with Nipped-B, a cohesin-loading factor that facilitates enhancer-promoter interactions, cohesins bind many activated enhancers but seem to preferentially associate with a subset of active genes linked to growth control and development [1]. This is consistent with findings that mutations in Nipped-B lead to Cornelia de Lange syndrome (CdLS), a genetic condition accompanied by developmental abnormalities and intellectual delay. The mechanism by which cohesin and Nipped B “choose” their gene targets from all the active genes has been elusive. A recent study by Swain et al. [2] provides important insights into this selection process. Using chromatin immunoprecipitations, followed by deep sequencing (ChIP-seq), bioinformatics, and binding studies, Swain et al. [2] identify two RNA-binding proteins, TBPH and Lark, that help guide the selection of genes bound by cohesin and Nipped-B. This is achieved by binding to nascent RNA transcripts and subsequent stabilization of cohesin and Nipped-B complexes on DNA (Fig 1).

TBPH is the *Drosophila* homolog of TAR DNA-binding protein (TARDP, or TDP-43), which harbors nuclear localization and export signals (NLS, NES), two RNA recognition motifs (RRMs), and a C terminus low complexity, prion-like domain [3]. It has been implicated in multiple aspects of gene expression, including transcription, splicing, mRNA transport, association with RNA stress granules (SGs), and translation [4]. Similar to TBPH, Lark, also known as RBM4, is an RNA-binding protein involved in splicing and translation regulation, comprising two RRM domains and a low complexity C terminus domain that are separated by a C2HC Zn finger-binding motif [5].

Multiple lines of evidence led the authors to hypothesize that RNA-binding proteins may help define the repertoire of genes bound by cohesin and Nipped-B complexes. First, TBPH/TDP-43 has been shown to bind UG repeats within its RNA targets [6–8], while Nipped-B associates preferentially with genes containing TG repeats downstream of transcription start sites [9]. Second, TDP-43 was found to regulate the transcription of the testis-specific mouse *acrv1* gene by binding to TGTGTG sequences within its promoter. Deletion of RRM1 or disabling RNA binding compromise TDP-43’s repressor function, suggesting that an RNA intermediate may be involved in its role as a transcriptional repressor [10]. Third, RNA immunoprecipitation experiments identified several transcripts produced by Nipped-B-bound genes as Lark targets [11]. Lark was also found to associate with transcripts of cohesin-bound genes by RNA affinity chromatography and mass spectrometry approaches [2].

To test this intriguing hypothesis, Swain et al. [2] used a ChIP-seq approach and found that cohesin, Nipped B, TBPH, and Lark bind genes and regulatory sequences such as enhancers and Polycomb Response Elements (PREs) in highly comparable patterns. This occurs both in cultured *Drosophila* cells and in wing epithelia, suggesting that these binding patterns are also present in vivo, in a developmental context. Next, the authors proceeded to decipher the mechanistic interactions between cohesion/Nipped-B and RNA-binding proteins using loss of function approaches. Depletion of TBPH by RNAi indicates that this RNA-binding protein facilitates the occupancy by cohesin and Nipped-B of most regulatory promoters, enhancers, and PREs with which they normally associate. In contrast, Lark appears to modify cohesin and Nipped-B binding sites differentially, depending on whether the sequences are contained within promoters, enhancers, or PREs. In the future, it will be interesting to determine what other molecular players may be mediating the complex effects of Lark on cohesin and Nipped-B and what the physiological consequences are of these seemingly differential interactions.

In keeping with these complexities, RNAi depletion studies indicate that Nipped-B also facilitates the binding of TBPH and Lark to genes and their regulatory sequences. This underscores the interdependency between cohesin/Nipped-B on one hand and RNA-binding proteins on another, and highlights an intricate interplay between these DNA- and RNA-binding proteins that will be important to uncover in future studies. Co-immunoprecipitation experiments from nuclear extracts indicate that these proteins form a complex driven by protein–protein interactions, independent of the presence of DNA or RNA. Furthermore, transcription is not required to maintain the association of Nipped-B, TBPH, and Lark with chromosomes. Together with in vitro RNA–protein binding studies, these findings support a scenario where TBPH and Lark interact with nascent RNAs generated from cohesin-binding genes and help stabilize Nipped-B, which in turn loads cohesin onto chromosomes. Although more work is needed in the future to determine the precise order of assembly, perhaps using live imaging studies, the data presented by Swain et al. [2] provides strong evidence for this model (Fig 1).

What does the future hold for the interplay between cohesin/Nipped-B- and RNA-binding proteins in regulating gene expression? The report highlighted here [2] opens up several new questions related to coordination of transcription and RNA processing during development, under stress and in disease (Fig 1). How might cohesin and Nipped-B aid RNA processing steps, including splicing, mRNA transport, and translation, that both TBPH/TDP-43 and Lark have been implicated in? It will be particularly interesting to determine the relationships between gene transcription and RNA processing controlled by TBPH/TDP-43 and Lark in a tissue-specific manner during development, as they may reveal novel mechanisms of human disease. TDP-43 is a DNA/RNA-binding protein linked to amyotrophic lateral sclerosis (ALS) and frontotemporal lobar degeneration (FTLD), two fatal neurodegenerative diseases [12]. Overwhelmingly, evidence points to depletion of TDP-43 from the nucleus and cytoplasmic accumulation as key factors in the pathomechanism of disease [13], therefore raising the possibility that misexpression of genes regulated by cohesin and Nipped-B could also play a role in neuronal death. While this seems paradoxical because of the fact that TDP-43 is involved in adult onset neurodegeneration, whereas Nipped-B mutations cause developmental delay, we note that TDP-43 phenotypes are modulated by factors required for development, including EphA4 [14] and Fragile X Mental Retardation Protein (FMRP) [15]. Interestingly, FMRP forms a functional complex with Lark and modulates circadian activity in *Drosophila* [16]. It will be interesting to determine whether these RNA-binding proteins share common RNA targets that may be under the control of cohesin and Nipped-B at the level of transcription. Given the involvement of TDP-43 in ALS/FTLD, Nipped-B in CdLS, and FMRP in the most common form of inherited mental retardation (Fragile X syndrome), these new findings raise the possibility that cognitive deficits and neuronal dysfunction in these conditions may share common molecular mechanisms whether they occur early or late in life. The future belongs to systems approaches expected to uncover new mechanisms by which DNA- and RNA-binding proteins give each other a helping hand in sculpting the landscape of gene expression regulation during development and in disease.
